# The increase in core body temperature in response to exertional-heat stress can predict exercise-induced gastrointestinal syndrome

**DOI:** 10.1080/23328940.2023.2213625

**Published:** 2023-05-24

**Authors:** Kayla Henningsen, Alice Mika, Rebekah Alcock, Stephanie K. Gaskell, Alexandra Parr, Christopher Rauch, Isabela Russo, Rhiannon M. J. Snipe, Ricardo J. S. Costa

**Affiliations:** a Department of Nutrition, Dietetics and Food, Monash University, Victoria, Australia; b Department of Dietetics and Human Nutrition, La Trobe University, Bundoora, Victoria, Australia; c Centre for Sport Research, School of Exercise and Nutrition Sciences, Deakin University, Burwood, Australia

**Keywords:** Exertional, heat, I-FABP, endotoxin, inflammatory cytokines, gastrointestinal symptoms, thermoregulation, endurance

## Abstract

Utilizing metadata from existing exertional and exertional-heat stress studies, the study aimed to determine if the exercise-associated increase in core body temperature can predict the change in exercise-induced gastrointestinal syndrome (EIGS) biomarkers and exercise-associated gastrointestinal symptoms (Ex-GIS). Endurance-trained individuals completed 2 h of running exercise in temperate (21.2-30.0°C) to hot (35.0-37.2°C) ambient conditions (n = 132 trials). Blood samples were collected pre- and post-exercise to determine the change in gastrointestinal integrity biomarkers and systemic inflammatory cytokines. Physiological and thermoregulatory strain variables were assessed every 10–15 min during exercise. The strength of the linear relationship between maximal (M-Tre) and change (Δ Tre) in rectal temperature and EIGS variables was determined via Spearman’s rank correlation coefficients. While the strength of prediction was determined via simple and multiple linear regression analyses dependent on screened EIGS and Ex-GIS confounding factors. Significant positive correlations between Tre maximum (M-Tre) and change (Δ Tre) with I-FABP (rs = 0.434, p < 0.001; and rs = 0.305, p < 0.001; respectively), sCD14 (rs = 0.358, p < 0.001; and rs = 0.362, p < 0.001), systemic inflammatory response profile (SIR-Profile) (p < 0.001), and total Ex-GIS (p < 0.05) were observed. M-Tre and Δ Tre significantly predicted (adjusted R2) magnitude of change in I-FABP (R2(2,123)=0.164, p < 0.001; and R2(2,119)=0.058, p = 0.011; respectively), sCD14 (R2(2,81)=0.249, p < 0.001; and R2(2,77)=0.214, p < 0.001), SIR-Profile (p < 0.001), and total Ex-GIS (p < 0.05). Strong to weak correlations were observed between M-Tre and Δ Tre with plasma concentrations of I-FABP, sCD14, SIR-Profile, and Ex-GIS in response to exercise. M-Tre and Δ Tre can predict the magnitude of these EIGS variables and Ex-GIS in response to exercise.

## Introduction

It is well established that exercise stress-induced perturbations to the gastrointestinal tract can compromise intestinal epithelial integrity as a result of reduced splanchnic perfusion and gastrointestinal tract targeted neuroendocrine activity [1]. Subsequently, translocation of luminal originating pathogenic agents, such as whole bacteria and bacterial endotoxins, is now well-recognized outcomes of such exercise-associated compromise to the integrity of the intestinal epithelium [2–4]. The persistence of luminal originating pathogenic translocation has the potential to stimulate both local and systemic inflammatory responses [5,6]. For example, bacterial endotoxins lipopolysaccharide and/or lipid A can easily permeate the intestinal epithelium into systemic circulation, with lipid A responsible for stimulating an initial innate systemic immune response [7]. These bacterial endotoxins that translocate into systemic circulation are deemed as foreign by extracellular and intracellular pattern recognition receptors, thus initiating a downstream inflammatory cascade [8]. Within controlled circumstances, this downstream inflammatory response is mediated by macrophages, monocytes, neutrophils, and natural killer cells that phagocytose and remove the foreign bodies responsible for the inflammatory cascade. However, due to unknown causes, the controlled inflammatory response may be disrupted and lead to ineffective removal of the foreign bodies and/or progression of infection, subsequently leading to clinical consequences that may potentially be fatal (e.g. sepsis and/or systemic shock) [2,3,8,9].

The occurrence and severity of compromised intestinal epithelial integrity and pathogenic translocation can be influenced by several extrinsic and intrinsic exacerbation factors during exercise, as previously described [5,6,10]. In short, extrinsic exacerbation factors may include the duration and intensity of exercise (i.e. the overall physiological strain), exercise modality, as well as the ambient conditions (i.e. temperature and humidity) during exercise [11–21]. Intrinsic exacerbation factors may include hydration status, age, biological sex, feeding tolerance, individual characteristics, such as fitness status, pre-disposition to diseases/disorders of the gastrointestinal tract, luminal and plasma bacterial and short chain fatty acid (SCFA) composition [4,22–27]. There is strong evidence, however, that ambient heat exposure during exercise, especially prolonged endurance-type exercise, results in the greatest disturbance to gastrointestinal status (i.e. epithelial injury, permeability, and lumen to circulation pathogenic translocation), and subsequent systemic immune responses [13,18–20,28], compared with other exacerbation factors. It has been postulated that the increased thermoregulatory (e.g. elevated core body temperature) and cardiovascular (e.g. elevated heart rate) strain during exercise in hot ambient conditions, compared with temperate ambient conditions, may exacerbate the primary causal pathways of exercise-induced gastrointestinal syndrome (EIGS) [6]. For example, 2 h of steady state exercise (i.e. running at ≥60% *V*O_2max_) in hot ambient conditions (i.e. ≥35°C) has consistently been shown to elicit peak core body temperatures ≥39.0°C and disturb all gastrointestinal biomarkers aligned with EIGS [6,10]. Whereas, exertional and exertional-heat stress models with insufficient exercise (e.g. <2 h exercise duration) and thermal (e.g. peak core body temperature ≤ 39.0°C) strain has presented with none to minimal pre- to post-exercise change in key EIGS biomarkers [29–31]. Therefore, research investigating the role of core body temperature as an exacerbation factor of EIGS that prompts sufficient exercise stress (e.g. ≥2 h at ≥60% *V*O_2max_) and thermoregulatory strain is warranted.

A previous systematic literature review explored the changes to gastrointestinal integrity biomarkers, namely targeting epithelial injury and permeability, in response to exertional and exertional-heat stress, with acknowledgment of core body temperature [32]. The outcomes generally suggested a positive relationship between the increase in core body temperature (i.e. pre- to post-exercise values) and biomarkers of intestinal epithelial integrity. However, no exploration of the magnitude of change on a comprehensive array of integrity biomarkers, systemic inflammatory cytokines, and exercise-associated gastrointestinal symptoms (Ex-GIS), in relation to maximal core body temperature and/or change in core body temperature was evident. Therefore, an apparent gap in the current exercise gastroenterology literature exists. From a translational professional practice perspective, EIGS endpoints, such as systemic inflammatory responses and Ex-GIS, are the prime factors linked to performance and clinical significance [2,3,9,23,33–36]. It is therefore important to understand the impact of the exacerbation effect of heat exposure during exercise, and its subsequent effect on core body temperature, on the full pathophysiological pathway spectrum of EIGS. In accordance with the background provisions, using available metadata, this study aimed to determine if absolute and relative increase in core body temperature, in response to prolonged endurance exercise with and without heat exposure, can predict the magnitude of response to EIGS biomarkers and Ex-GIS. These biomarkers include plasma concentrations of intestinal fatty acid-binding protein (I-FABP), soluble CD14 (sCD14), lipopolysaccharide-binding protein (LBP), endotoxin core antibody immunoglobulin (Ig) M, systemic inflammatory cytokines (i.e. TNFα, IL-1β, IL-6, IL-8, IL-10, and IL-1ra), and systemic inflammatory response profile (SIR-Profile). It was hypothesized that the increase in core body temperature in response to exertional and/or exertional-heat stress would proportionally result in the increase in gastrointestinal integrity biomarkers, systemic inflammatory responses, and Ex-GIS.

## Methods

### Participants

Metadata from 12 experimental trials, within 9 previously published research studies, that utilized an exertional or exertional-heat stress exercise model (i.e. 2 h of running exercise in an ambient-controlled environment) were used for this exploratory data analyses [13,15–20,28,37]. One hundred and thirty-two trials were included from endurance-trained individuals [mean ± SD (male *n* = 97, female *n* = 35): age 32.1 ± 7.7 years, nude body mass 70.8 ± 10.8 kg, height 1.70 ± 0.28 m, % body fat mass 15.5 ± 5.8, *V*O_2max_ 57.7 ± 7.9 mL·kgBM^−1^·min^−1^, training load 457 ± 245 min·week^−1^ (i.e. running (endurance and ultra-endurance), cycling, swimming, CrossFit, triathlon, and/or team sports)] that volunteered to participate in the respective experimental trials. The female participants completed the experimental trials during the early-mid follicular phase of the menstrual cycle. All participants provided written informed consent prior to the initiation of any experimental activity, which was approved from the local Monash University Human Research Ethics Committee and conformed to the 2013 Helsinki Declaration for Human Research Ethics (Suplementary Table S1). The standard inclusion criteria were non-heat acclimated male or female endurance running trained individuals, that were absent of illness and/or disease, and aged between 18 and 50 years. Participants were excluded if they confirmed having gastrointestinal infections, diseases, and/or disorders (e.g. celiac disease, inflammatory bowel disease, irritable bowel syndrome, diverticular disease, gastro-esophageal reflux disease, past history of gastrointestinal surgery, and/or other self-reported gastrointestinal issues), consumed potential modifiers of gastrointestinal integrity (e.g. prebiotics, probiotics, and/or antibiotics), adhered to gastrointestinal-focused dietary regimes (e.g. low fermentable oligo, di-, mono-saccharide, and polyol (FODMAP) and/or fiber-modified diets) within the previous 3 months, or consumed nonsteroidal anti-inflammatory medications and/or stool altering medications (e.g. laxatives and antidiarrheal) within 1 month before the experimental protocol.

### Preliminary measures

Approximately 1 week prior to the control trial, each participant’s *V*O_2max_ was measured via a continuous incremental exercise test to volitional exhaustion on a motorized treadmill, as previous description [38]. Each participant’s running speed for the experimental trial was determined by extrapolating and verifying the data from the *V*O_2_ work rate relationship. Participants were provided with a low fermentable oligo, di-, mono-saccharide, and polyol (FODMAP) diet for 24 h prior to the experimental trial to reduce any confounding gastrointestinal symptom issues that may arise from the lead-in diet [13]. Each participant’s pre-trial dietary intake varied based on their individual energy and macronutrient requirements (7.7–14.4 MJ, 266–530 g carbohydrate, 68–124 g protein, 51–85 g fat). No strenuous exercise was performed by the participants for 48 h prior to the experimental trial. Compliance was confirmed with a dietary and exercise record. The participants presented at the laboratory at 08:00 h after consumption of the standardized low FODMAP breakfast at 07:00 h (2.5 MJ, 125 g carbohydrate, 14 g protein, and 4 g fat) with 400 mL of water.

### Experimental procedure

Each participant completed a 2 h exercise protocol, consisting of running at steady state at 60% *V*O_2max_ [13,18–20,28,37], or a high-intensity interval ranging between ~55% to 80% *V*O_2max_ [15–17], in ambient temperatures (T_amb_) ranging from 21.2°C to 37.5°C and 20.1% to 50.0% relative humidity (RH), with a fan air speed at fan airspeed ~10.6 km·h^−1^. Core body temperature was determined through rectal temperature (T_re_) and gastrointestinal symptoms were recorded every 10–15 min during the exercise trial. Rectal temperature was monitored via a thermocouple inserted 12 cm beyond the external anal sphincter. The athletes Ex-GIS were recorded via an exercise-specific gastrointestinal symptom assessment tool (modified visual analog scale (mVAS)) [33,39]. In short, participants were educated and advised to complete the rating scale as follows: 1–4 indicative of mild GIS (i.e. sensation of GIS, but not substantial enough to interfere with exercise workload) and increasing in magnitude, 5–9 indicative of severe GIS (i.e. GIS substantial enough to interfere with exercise workload), and 10 indicative of extremely severe GIS warranting exercise cessation. If no specific GIS was reported, this was indicative of 0, and subsequently no rating was warranted. GIS were specified and categorized into upper-GIS (i.e. gastro-esophageal: belching, gastric bloating, upper abdominal pain, urge to regurgitate, and/or regurgitation), lower-GIS (i.e. intestinal: flatulence, lower abdominal bloating, lower abdominal pain, urge to defecate, and/or abnormal defecation), and others (i.e. acute transient abdominal pain (stitch), dizziness, and nausea). Blood samples, via venepuncture, were collected pre-exercise, immediately post-exercise, 1 h and 2 h post-exercise into lithium heparin (6 mL, 1.5 IU·mL^−1^ heparin), and EDTA (4 mL, 1.6 mg·mL^−1^ K3EDTA) vacutainers (BD, Oxford, UK). The whole blood was centrifuged at 1500 g for 10 min within 15 min of sample collection.

The plasma was aliquoted and frozen at −80°C until analysis was performed. The plasma concentrations of I-FABP (HK406; Hycult Biotech, Uden, the Netherlands) a biomarker indicative of intestinal epithelial cell damage, LBP (HK315; Hycult Biotech) a surrogate biomarker indicating the translocation of lipopolysaccharide specifically from the intestinal lumen to systemic circulation, sCD14 (HK320; Hycult Biotech) a surrogate biomarker indicating the broad-spectrum translocation of bacterial endotoxins from the intestinal lumen to systemic circulation, and endogenous endotoxin core antibody IgM (HK504; Hycult Biotech) were determined via ELISA [10]. The plasma concentrations of systemic inflammatory cytokines TNFα, IL-1β, IL-6, IL-8, IL-10 and IL-1ra (HCYTOMAG-28SK; MilliporeSigma, Darmstadt, Germany) were assessed via multiplex system. Analytical procedures and assay coefficient of variation are reported within the original research publication [13,15–20,28,37]. Considering the large intra- and inter-individual variation in inflammatory cytokine responses previously observed, the peak Δ pre- to post-exercise for pro-inflammatory (IL-1β and TNF-α), response/modulatory (IL-6 and IL-8) and anti-inflammatory (IL-10 and IL-1ra) cytokines were combined to establish an exercise-associated systemic inflammatory response profile (SIR-Profile) for comparative purposes, as previously described [21]. In short, absolute peak post-exercise plasma cytokine concentrations in pg/mL (i.e. TNFα, IL-1β, IL-6, IL-8, IL-10 and IL-1ra) were subtracted from absolute baseline pre-exercise plasma concentrations in pg/mL, to establish the magnitude of concentration change in pg/mL. These values were then summed to provide an arbitrary unit (arb.unit) indicative of overall systemic inflammation; whereby, incorporating the generally classified pro-, response/modulatory-, and anti-inflammatory cytokines [10].

### Statistical analysis

The sample size of each included experimental study [13,18–20,28,37] had previously been calculated *a priori* and reported to provide sufficient sample size for statistical precision. In addition, population size for current data analysis was checked and confirmed post hoc (G*Power 3.1, Kiel, Germany), using α 0.05 and beta β 0.80 with correlation and linear multi-regression models. Included metadata from experimental trials did not provide full participant numbers for all indicated EIGS biomarkers; therefore, sample size numbers for each respective EIGS biomarker and Ex-GIS are presented within. Data in the text and tables are presented as either mean ± SD or mean and 95% confidence interval (CI), as indicated; and total accumulative score and 95% CI for Ex-GIS, as previously reported (Gaskell et al., 2019). The data presented within the figures demonstrate the trend of significant results. Prior to two-tailed correlation analyses, diagnostic checks on normality (Kolmogorov–Smirnov test of normality) were performed. Spearman’s rank correlation coefficients were utilized to determine the strength of the linear relationship between maximum rectal temperature (M-T_re_) and rectal temperature change (Δ T_re_) with gastrointestinal integrity biomarkers, systemic inflammatory cytokines, and Ex-GIS. The strength of the linear relationship between the variables and the prediction of change in the gastrointestinal integrity biomarkers and cytokines were determined as very weak at <.200, weak at .200–.399, moderate at .400–.599, and strong at ≥.600. Simple linear regression analyses were used to determine if M-T_re_ and Δ T_re_ can predict changes in the gastrointestinal integrity biomarkers, systemic inflammatory cytokines, and Ex-GIS of individuals reporting Ex-GIS. In order to meet the assumption of normality required for regression analysis, data were log transformed, outliers identified via box plot and removed, and this transformed data were used in the regression analysis. In addition, confounding factors [i.e. biological sex, age, fitness status (*V*O_2max_), hydration status (plasma osmolality), and during exercise variation in heart rate (i.e. initial vs end-point steady state exercise)], previously reported or suggested to exacerbate primary analysis variables [18,22–24,27], were screened to ascertain relationship with the current data set. Identified significant relationships were then employed by applying multiple regression analyses. Statistical analyses were performed via SPSS (V.28.0, Chicago Illinois, USA) with statistical significance accepted at *p* < 0.05.

## Results

Exertional (*n* = 73) and exertional-heat stress (*n* = 59) mean pre-exercise T_re_, M-T_re_ and Δ T_re_ were 36.98°C (36.91°C to 37.04°C), 38.60°C (38.47°C to 38.74°C), and 1.70°C (1.57°C to 1.82°C), respectively. A strong correlation was observed between M-T_re_ and Δ T_re_ in response to the exertional and exertional-heat stress trials (*r*_s_= .757, *n* = 132, *p* < 0.001). There was a moderate significant positive relationship between M-T_re_ and Δ T_re_ with ambient conditions at which the exertional (T_amb_ 24.2°C (23.6°C to 24.8°C) and 42% (41% to 44%) RH) or exertional-heat (T_amb_ 35.1°C (34.9°C to 35.3°C) and 29% (27% to 31%) RH) stress the exercise was performed (*r*_s_= .559, *n* = 132, *p* < 0.001, and *r*_s_= .505, *n* = 132, *p* < 0.001, respectively).

**Figure 1.**
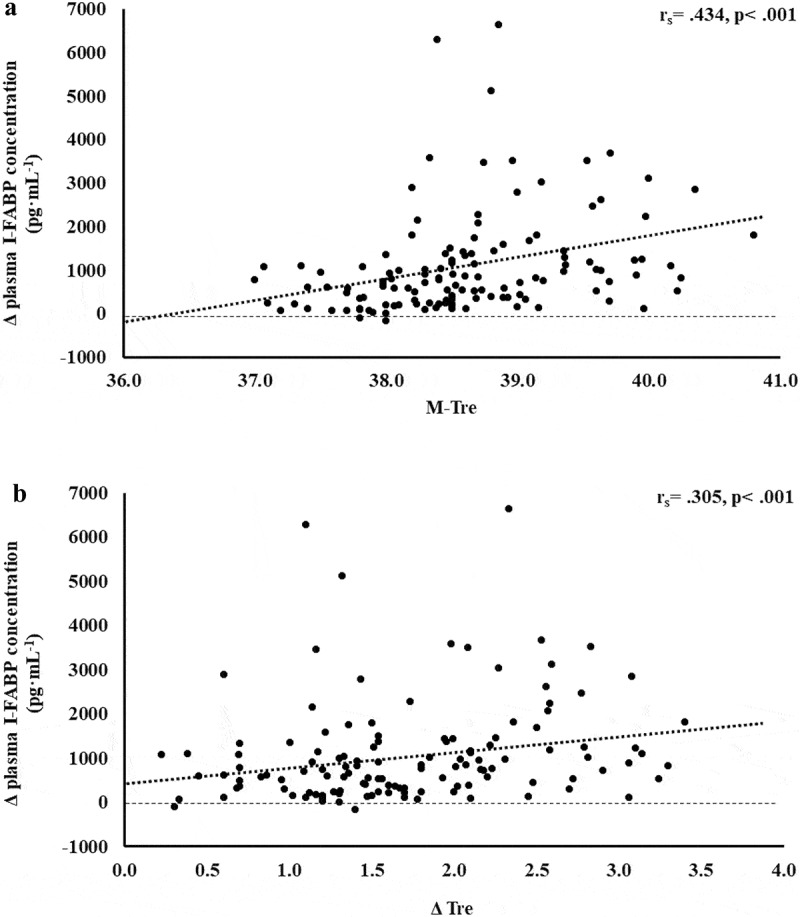
Association between M-T_re_ (a) and Δ T_re_ (b) with plasma concentrations of I-FABP in response to 2 h of strenuous running exercise in temperate (20–30°C) and hot (~35°C) ambient conditions.

**Figure 2.**
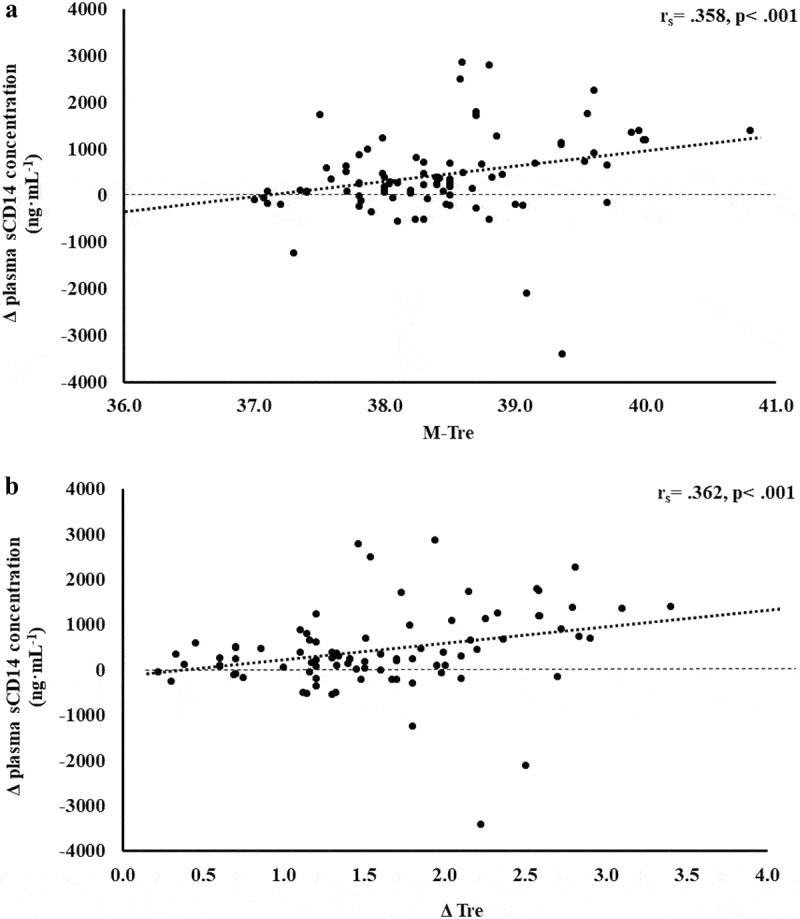
Association between M-T_re_ (a) and Δ T_re_ (b) with plasma concentrations of sCD14 in response to 2 h of strenuous running exercise in temperate (20–30°C) and hot (~35°C) ambient conditions.

**Figure 3.**
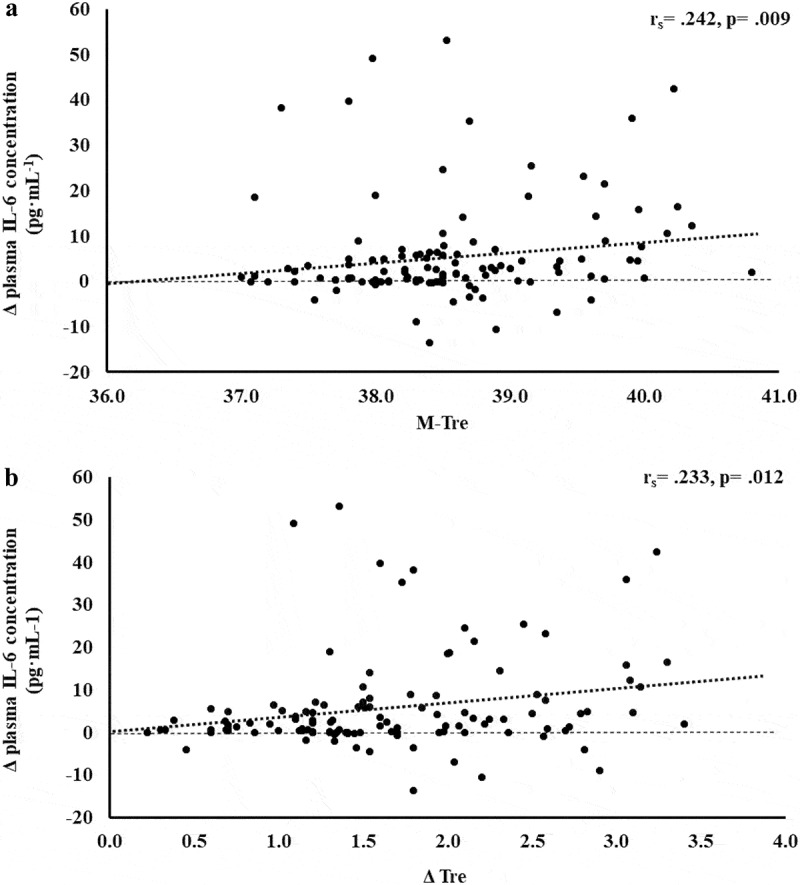
Association between M-T_re_ (a) and Δ T_re_ (b) with plasma concentrations of IL-6 in response to 2 h of strenuous running exercise in temperate (20–30°C) and hot (~35°C) ambient conditions.

**Figure 4.**
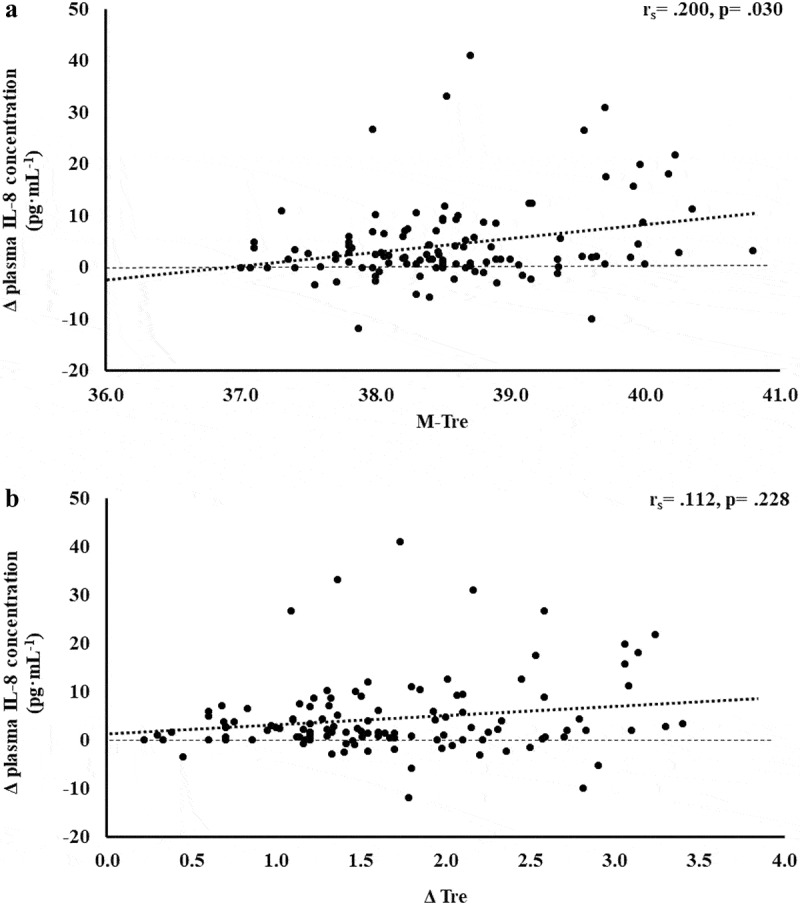
Association between M-T_re_ (a) and Δ T_re_ (b) with plasma concentrations of IL-8 in response to 2 h of strenuous running exercise in temperate (20–30°C) and hot (~35°C) ambient conditions.

**Figure 5.**
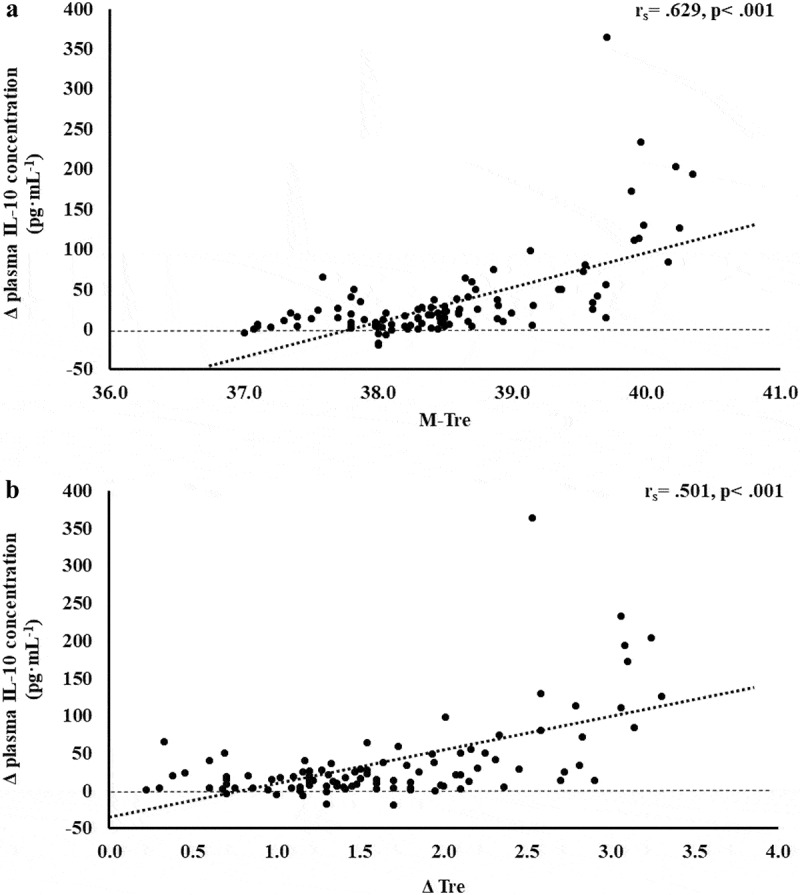
Association between M-T_re_ (a) and Δ T_re_ (b) with plasma concentrations of IL-10 in response to 2 h of strenuous running exercise in temperate (20–30°C) and hot (~35°C) ambient conditions.

**Figure 6.**
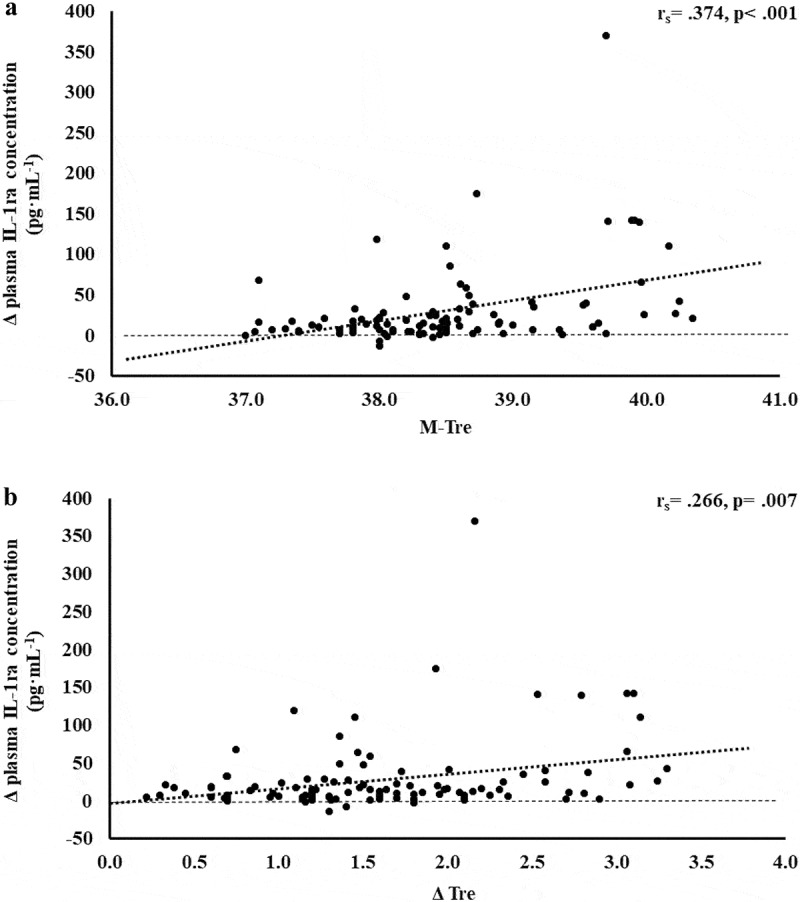
Association between M-T_re_ (a) and Δ T_re_ (b) with plasma concentrations of IL-1ra in response to 2 h of strenuous running exercise in temperate (20–30°C) and hot (~35°C) ambient conditions.

**Figure 7.**
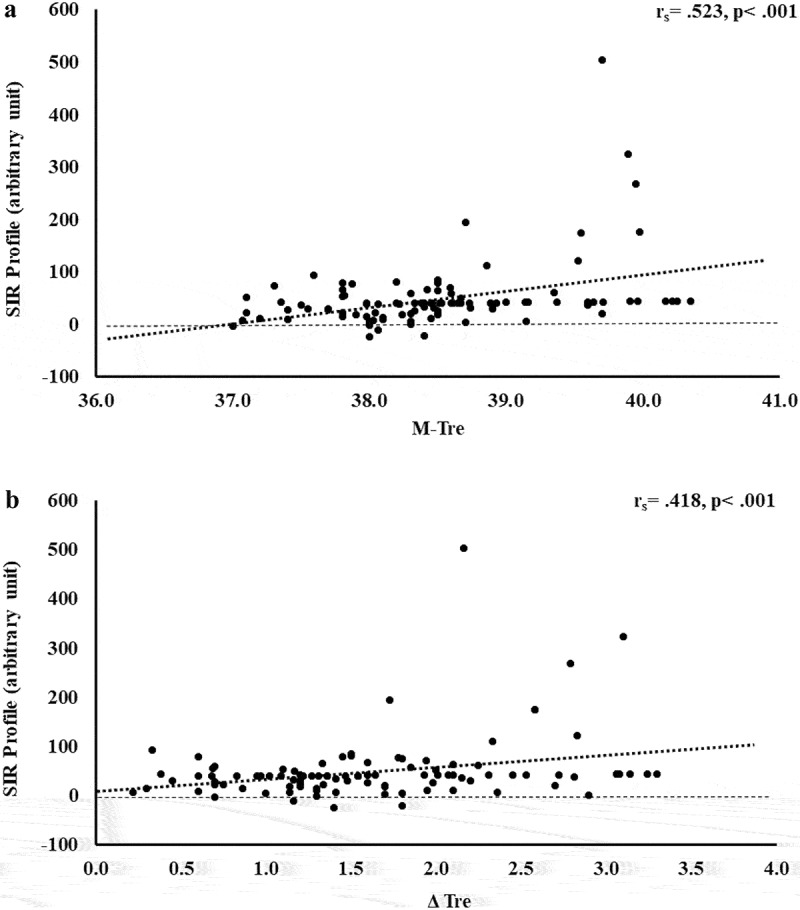
Association between M-T_re_ (a) and Δ T_re_ (b) with plasma concentrations of SIR-Profile in response to 2 h of strenuous running exercise in temperate (20–30°C) and hot (~35°C) ambient conditions.

The results of correlation analyses that determined the strength of the relationship between the M-T_re_ and Δ T_re_ with IFABP, LBP, sCD14, IgM, TNFa, IL-1b, IL-6, IL-8, IL-10, IL-1ra, and SIR are displayed in Table 1. Significant positive relationships between, both or either, M-T_re_ and Δ T_re_ with pre- to post-exercise change in plasma concentrations of I-FABP (Figure 1), sCD14 (Figure 2), IL-6 (Figure 3), IL-8 (Figure 4), IL-10 (Figure 5), IL-1ra (Figure 6), and SIR profile (Figure 7) were observed.

**Figure 8.**
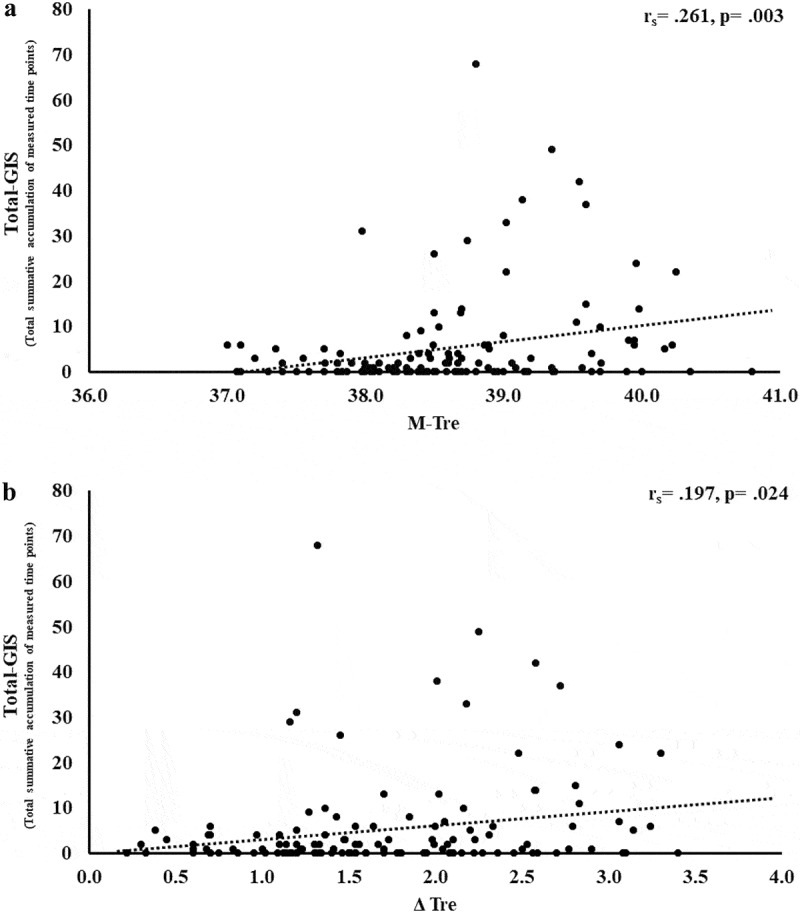
Association between M-T_re_ (a) and Δ T_re_ (b) with total exercise-associated gastrointestinal symptoms in response to 2 h of strenuous running exercise in temperate (20–30°C) and hot (~35°C) ambient conditions.

**Table 1.** Association between M-T_re_ and Δ T_re_ with plasma concentrations of EIGS epithelial integrity and systemic inflammatory biomarkers in response to 2 h of strenuous running exercise in temperate (20–30°C) and hot (~35°C) ambient conditions.

		Mean	95% CI Lower	Upper	*n*	M-T_re_ r_s_	*p*	Δ T_re_ r_s_	*p*
I-FABP	(pg·mL^−1^)	1083	881	1285	128	.**430**	**<.001**	.**305**	**<.001**
LBP	(ng·mL^−1^)	1573	953	2194	83	.040	.720	.148	.183
sCD14	(ng·mL^−1^)	400	213	586	87	.**358**	**<.001**	.**362**	**<.001**
IgM	(MMU·mL^−1^)	26.0	11.4	40.6	88	.011	.921	.031	.776
TNFα	(pg·mL^−1^)	3.3	1.9	4.6	117	.030	.746	.046	.625
IL-1β	(pg·mL^−1^)	1.3	0.5	2.1	117	.002	.979	.007	.941
IL-6	(pg·mL^−1^)	5.8	3.8	7.8	117	.**242**	.**009**	.**233**	.**012**
IL-8	(pg·mL^−1^)	4.5	3.0	5.9	117	.**200**	.**030**	.112	.228
IL-10	(pg·mL^−1^)	35.0	24.3	45.8	102	.**629**	**<.001**	.**501**	**<.001**
IL-1ra	(pg·mL^−1^)	29.4	19.8	39.0	102	.**374**	**<.001**	.**266**	.**007**
SIR Profile	(arb.units)	76.7	59.2	99.9	102	.**523**	**<.001**	.**418**	**<.001**

The results of correlation analyses that determined the strength of the relationship between the M-T_re_ and Δ T_re_ with total-GIS, upper-GIS, lower-GIS, and nausea are displayed in Table 2. Significant positive relationships between M-T_re_ and Δ T_re_ with total-GIS, upper-GIS, and lower-GIS were observed.

**Table 2.** Association between M-T_re_ and Δ T_re_ with exercise-associated gastrointestinal symptoms (Ex-GIS) in response to 2 h of strenuous running exercise in temperate (20–30°C) and hot (~35°C) ambient conditions.

	Mean^#^	Individual responses (lower and upper)^##^		*n*	M-T_re_ r_s_	*p*	Δ T_re_ r_s_	*p*
Total-GIS	5	1	68	131	.**261**	.**003**	.**197**	.**024**
Upper-GIS	2	1	36	131	.**384**	**<.001**	.**269**	.**002**
Lower-GIS	1	1	31	131	.**173**	.**048**	.**175**	.**046**
Nausea	1	1	16	131	.105	.232	.037	.673

^#^Summative accumulation of measured time points, ^##^ individual range of participants reporting incidence of GIS.

Outcomes of simple and multiple-regression analyses are presented in Table 3. Analyses outcomes showed that increases in M-T_re_ and Δ T_re_ significantly predicted increases in plasma I-FABP concentration (*β* = 0.369, *p* < 0.001; and *β* = 0.206, *p* = 0.031, respectively); however, variation in heart rate during exercise (i.e. cardiovascular strain) as a confounding variable did not significantly predict increases or variations in I-FABP (*β* = 0.112, *p* = 0.183; and *β* = 0.118, *p* = 0.214, respectively). Increases in M-T_re_ and Δ T_re_ significantly predicted increases in plasma sCD14 concentration (*β* = 0.406, *p* < 0.001; and *β* = 0.373, *p* < 0.001, respectively); but fitness status as a confounding variable also provided some variation and predictive effect for sCD14 levels (*β* = 0.277, *p* = 0.005; and *β* = 0.289, *p* = 0.005, respectively). From a systemic inflammatory response perspective, increases in M-T_re_ and Δ T_re_ significantly predicted increases in plasma IL-10 (*β* = 0.645, *p* < 0.001; and *β* = 0.534, *p* < 0.001, respectively) and IL-1ra (*β* = 0.369, *p* < 0.001; and *β* = 0.219, *p* = 0.041, respectively) concentration, and the SIR-Profile (*β* = 0.560, *p* < 0.001; and *β* = 0.433, *p* < 0.001, respectively). Significance was not observed for during exercise variation in heart rate during exercise as a confounding factor for IL-10 (*β* = 0.098, *p* = 0.211; and β = 0.020, *p* = 0.834, respectively) and SIR-Profile (*β* = 0.069, *p* = 0.421; and *β* = 0.014, *p* = 0.892, respectively). From an Ex-GIS perspective, increases in M-T_re_ and Δ T_re_ significantly predicted increases in total- (*β* = 0.368, *p* = 0.001; and *β* = 0.341, *p* = 0.003, respectively) and upper- (*β* = 0.371, *p* = 0.011; and *β* = 0.354, *p* = 0.017, respectively) GIS. No significant outcomes in regression analyses were observed for plasma TNFα, IL-1β, LBP and IgM concentration, and lower-GIS and nausea (Table 3). Due to lack of normality, regression analyses for plasma IL-6 and IL-8 concentrations were not performed.

**Table 3.** Magnitude of prediction of change in plasma concentrations of EIGS epithelial integrity, systemic inflammatory biomarkers, and exercise-associated gastrointestinal symptoms (Ex-GIS) in response to 2 h of strenuous running exercise in temperature (20–30°C) and hot (~35°C) ambient conditions.

	Adjusted R^2^	F	df	*p*
**M-Tre**				
I-FABP	.164	12.0	2, 123^a^	**<.001**
LBP	−.012	0.1	1, 78	.774
sCD14	.249	14.7	2, 81^b^	**<.001**
IgM	−.012	0.0	1, 81	.925
TNFα	−.019	0.1	2, 97^a^	.932
IL-1β	−.017	2.5	1, 83	.119
IL-10	.424	35.9	2, 93^a^	**<.001**
IL-1ra	.126	14.2	1, 90	**<.001**
SIR Profile	.312	22.8	2, 94^a^	**<.001**
Total-GIS	.124	11.6	1, 74	**<.001**
Upper-GIS	.118	7.0	1, 44	.**011**
Lower-GIS	−.032	0.1	1, 29	.787
Nausea	.191	2.9	2, 14^c^	.089
**Δ T**_**re**_				
I-FABP	.058	4.7	2, 119^a^	.**011**
LBP	−.001	1.0	1, 75	.310
sCD14	.214	11.8	2, 77^b^	**<.001**
IgM	−.011	0.1	1, 81	.756
TNFα	−.020	0.1	2, 93^a^	.947
IL-1β	−.019	2.5	1, 80	.116
IL-10	.263	17.1	2, 88^a^	**<.001**
IL-1ra	.037	4.3	1, 85	.**041**
SIR Profile	.166	10.0	2, 89^a^	**<.001**
Total-GIS	.104	9.4	1, 71	.**003**
Upper-GIS	.105	6.2	1, 43	.**017**
Lower-GIS	−.034	0.0	1, 29	.957
Nausea	.132	2.1	2, 13^c^	.157

^a^multiple regression analysis with variation in heart rate during exercise-confounding factor, ^b^multiple regression analysis with fitness status (*VO_2_max*) confounding factor, and ^c^multiple regression analysis with hydration status (plasma osmolality) confounding factor.

## Discussion

Using available metadata, the current study aimed to determine if an increase in core body temperature, as measured by rectal temperature, in response to prolonged endurance exercise with and without heat exposure can predict the response magnitude of EIGS biomarkers and Ex-GIS. In accordance with our hypothesis, the increase in absolute maximal and relative change in core body temperature, in response to prolonged strenuous exercise with and without heat exposure, showed a strong to weak positive correlation with plasma I-FABP, sCD14, a cluster of systemic inflammatory cytokines (i.e. IL-6, IL-8, IL-10, and IL-1ra), and Ex-GIS (i.e. total-, upper-, and lower-GIS). Absolute maximal core body temperature presented a higher correlation values in the majority of measured EIGS biomarkers and Ex-GIS, compared with relative change in core body temperature. On the contrary to our hypothesis, no correlations were observed between core body temperature measures with plasma concentrations of LBP, IgM, IL-1β, and TNFα, and the Ex-GIS nausea. In addition, regression analyses showed that the increase in absolute maximal and relative change in core body temperature, as a result of exertional or exertional-heat stress, can predict the magnitude of rise in plasma concentrations of I-FABP, sCD14, systemic anti-inflammatory cytokines IL-10 and IL-1ra, total and upper Ex-GIS. Similarly, absolute maximal core body temperature consistently presenting higher predictive values compared with relative change in core body temperature. These findings suggest that elevations in core body temperature during exercise, exacerbated by exposure to hot ambient conditions, can create significant perturbations to gastrointestinal epithelial integrity, systemic inflammatory responses, and Ex-GIS, namely upper-GIS.

Released from intestinal enterocytes into circulation in response to cell damage, I-FABP is a common surrogate biomarker for detecting intestinal epithelial injury in response to stimuli that promotes perturbation to cellular stability [10,40,41]. The current study reports that the thermoregulatory strain, indicated by increases in absolute maximal and relative change in core body temperature, results in a moderate positive relationship with plasma I-FABP concentration. The total variation in plasma I-FABP concentration can be explained by M-T_re_ and Δ T_re_, with 16.4% and 5.8% significant contributions (adjusted R^2^), respectively. Moreover, bearing in mind the comprehensive list of extrinsic and intrinsic exacerbation factors of EIGS and Ex-GIS previously reported [10], the adjunct analysis of cardiovascular strain (i.e. variation in heart rate from initial- to end-stage steady state), as an identified confounding factor during the screening process, provided insignificant contribution. Using data from the current study, it is estimated that a mild increase in core body temperature of ≤39.0°C, in response to exertional or exertional-heat stress, would result in Δ pre- to post-exercise of ≤1200 pg·mL^−1^ in plasma I-FABP concentrations. While, more substantial thermoregulatory strain (i.e. core body temperature ≥39.5°C) would result in ≥1500 pg·mL^−1^, which is in accordance with a recent EIGS biomarker reliability study proposing 1301 pg·mL^−1^ for relevant minimal detectable change [4], and indicative of similar to higher values observed in clinical populations aligned with diseases/disorders of the gastrointestinal tract [34,42–44]. These results are not surprising considering a substantial amount of exertional and exertional heat stress studies that have employed the I-FABP biomarker have consistently shown higher absolute and relative change concentration after exercise in hot ambient conditions (i.e. ~35°C) compared with temperate ambient conditions [13,15–20,24]. These correlative and predictive outcomes are likely explained by exertional-heat stress promoting greater splanchnic hypoperfusion (i.e. greater blood redistribution to peripheral circulation to aid thermoregulation and internal heat dispersion) [45–47], and/or increased splanchnic temperature possibly evoking damage and/or rupture to the enterocyte cellular membrane [48,49]. It is also noteworthy to report that these thermoregulatory linked significant contributing factors can only partly explain the EIGS pathophysiology of increased intestinal epithelial cell injury. It is likely that other recognized extrinsic (e.g. exercise duration, intensity, and/or modality) and intrinsic (e.g. biological sex, fitness status, hydration status) exacerbation factors, although not contributing a substantial amount as identified in the screening process of data analyses, in combination may provide considerable stimuli for the outcomes observed. From a professional practice perspective, directly applying pre- and/or during-exercise strategies to attenuate the increase in core body temperature during exercise, and avoiding the critical elevated core body temperature set-point (e.g. ≥39.5°C), in adjunct with direct or indirect management of other exacerbation factors, may reduce the exercise-associated injury to the intestinal epithelium, and mitigate any possible clinical episodes (e.g. acute reversible colitis and associated GIS).

Several surrogate biomarkers have been proposed to represent and provide indication of translocation of luminal originating pathogenic bacterial endotoxins into circulation; namely sCD14, LBP, and endogenous endotoxin core antibodies (EndoCAb) [10,50]. Outcomes of this study suggest no correlation and predictive power between M-T_re_ and Δ T_re_ with plasma concentrations of LBP and EndoCAb IgM. However, plasma sCD14 concentration showed moderate positive correlations with M-T_re_ and Δ T_re_. The total variation in plasma sCD14 concentration can be explained by M-T_re_ and Δ T_re_, with 24.9% and 21.4% significant contributions, respectively; in conjunction with significant correlation and prediction contribution from fitness status (i.e. *V*O_2max_) as a confounding factor. Using data from the current study, it is estimated that a mild increase in core body temperature of ≤39.0°C, in response to exertional or exertional-heat stress, would result in Δ pre- to post-exercise of ≤600 ng·mL^−1^ in plasma sCD14 concentrations. While more substantial thermoregulatory strain (i.e. core body temperature ≥39.5°C) would result in Δ pre- to post-exercise ≥800 ng·mL^−1^, which is in accordance with a recent EIGS biomarker reliability study proposing ≥780 ng·mL^−1^for relevant minimal detectable change [4]. Such outcomes are in accordance with a recent murine experimental model, whereby Wister rats were subjected to 90 min treadmill exercise (15 m·min^−1^) in 13°C, 24°C, or 31°C ambient conditions, versus a 24°C rest control [51]. Compared with rest and exercising at the lower ambient temperatures, exercise in 31°C resulted in rats producing greater core temperature rises (e.g. >40°C), as measured via telemetry. These thermal strain outcomes subsequently resulted in greater increases in intestinal permeability measured via a radioactive probe method and also increased tight-junction disturbance, as measured by mRNA occludin and zonula occludens-1 gene expression. It was reported that the increase in permeability and tight-junction protein gene expression were positively associated with the magnitude of core temperature. It is, however, important to highlight that such experimentation measuring intestinal permeability and/or tight-junction status can only provide inference on pathogenic translocation, as it is only considered a gateway marker and not objectively detecting the lumen to circulation translocation of pathogenic agents [10]. Reverting back to the current study and human experimental models, considering correlations and predictions between core body temperature values and plasma sCD14 concentration were evident, but not for LBP and EndoCAb IgM, this suggests sCD14 appears to be a more sensitive EIGS biomarker than LBP and IgM when conducting gastroenterology research that is focused on exploration or intervention in modifying core body temperature. Results also suggest that fitness status, in conjunction with core body temperature, provided significant contribution to plasma sCD14 concentration in response to exercise with or without ambient heat exposure. These outcomes are in accordance with previous exertional and exertional-heat stress studies that report substantial exercise-associated endotoxemia in athletes with a high fitness status [2,3]; but contrary to another study investigating untrained versus trained participants on LPS translocation in response to exertional-heat stress (i.e. 106 min walking at 4.5 km·h^−1^ in 40°C) [52]. Any clinical concern of such enhanced endotoxemia with higher fitness status is supplemented by potential enhanced anti-endotoxin antibody responses (i.e. endotoxin neutralization and clearance capacity) and compensatory anti-inflammatory responses (i.e. counteracting the systemic inflammatory response) seen in highly trained individuals [2,3,50,53–56]. However, this may become of greater concern in those of lower fitness status and “not fit for task,” whereby compensatory responses are not as developed and effective.

Within EIGS pathophysiology, systemic inflammatory responses are the profound outcome of luminal-originating pathogen translocation into systemic circulation [10]. Such EIGS downstream outcomes may be asymptomatic and transient in nature, or can lead to a fatality, especially when exercising in hot ambient conditions (i.e. systemic inflammatory response syndrome and multi-organ failure as a result of sepsis) [2,3,9,36]. Without discounting and having appreciation for the additional potential for translocation of luminal originating digestive enzymes that has been discussed in relation to endogenous tissue damage and failure [57,58]. It is an important aspect of professional practice to understand how core body temperature impacts key systemic inflammatory cytokines linked with clinical outcomes [59]. The current study’s outcomes show no correlation and/or predictive power between M-T_re_ and Δ T_re_ with plasma concentrations of proinflammatory cytokines IL-1β and TNF-α. This is likely due to the repeatable observation that laboratory-controlled experimental exercise protocols fail to elicit a pro-inflammatory response in the majority of study participants, linked to either the insufficient exertional or exertional-heat stress, and/or competent compensatory anti-inflammatory responses to the modest exertional or exertional-heat stress [10]. This contrasts with findings from field-based studies with greater exertional and/or heat stress that have reported exercise-associated increases in plasma IL-1β and/or TNF-α concentrations [2,3,53–55,60]. In addition, the limited endotoxemia (e.g. mean Δ: sCD14 400 ng·mL^−1^, LBP: 1573 ng·mL^−1^, and IgM 26 MMU·mL^−1^) observed in the current metadata set may be reflected in the current study’s insignificant relationship between M-T_re_ and Δ T_re_ with plasma concentrations of TNFα and IL-1β, since the activation of these cytokines relies on pathogen presence (e.g. LPS and subsequent LBP and sCD14 activation) [10,61,62]. Plasma concentrations of inflammatory response or modulatory cytokines IL-6 and IL-8 showed lower-end moderate positive correlations with M-T_re_ and Δ T_re_. However, due to the commonly observed individual variation in these cytokines, poor test–retest reliability, and consistent high values in certain individual study participants [4,10,15–17], a large number of outliers were identified, normality was not achieved, and regression analysis was not possible. The most notable systemic inflammatory cytokine findings were the strong-moderate positive correlation between anti-inflammatory cytokines IL-10 and IL-1ra with M-T_re_ and Δ T_re_. The total variation in plasma IL-10 concentration can be explained by M-T_re_ and Δ T_re_, with 42.4% and 26.3% significant contributions, respectively. The adjunct analysis of cardiovascular strain as an identified confounding factor during the screening process provided insignificant contribution, and the total variation in plasma IL-1ra concentration can be explained by M-T_re_ with 12.6% and 3.7% significant contributions. Findings from the current study suggest increases in core body temperature in response to exercise with or without heat exposure will increase anti-inflammatory responses, namely IL-10. This supports the prime measurement of plasma IL-10 concentration over other commonly used inflammatory cytokine markers in assessing the impact of core body temperature on systemic inflammatory responses during exertional and/or exertional-heat stress experimental models.

A prime observation in the current metadata analyses appears to be a compensatory anti-inflammatory response haltering any pathological increases in key proinflammatory cytokines, namely TNFα and IL-1β, that are also considered pyrogenic cytokines, and contribute to internal heat gains through promoting the febrile response [2,3,63,64]. Although in the current study’s metadata set low pre- to post-exercise TNFα and IL-1β responses were observed, in situation of greater pyrogenic proinflammation (e.g. endurance and ultra-endurance activities in the heat within excessive training tasks, competition, or occupation settings, with predisposing factors: compromised immunity, “not fit for task,” equipment and/or anthropometry effecting thermoregulation), it is plausible that compromised anti-inflammation would prevail [2,3,55,59,60,65,66]. This may lead to a spiral-effect, whereby external heat exposure (i.e. ambient conditions, and/or direct or indirect heat exposure) and internal heat production (i.e. skeletal muscle contraction, metabolic kinetics, and/or febrile responses) summate to override body’s thermoregulatory capacity, potentially leading to fatality (e.g. heat stroke pathophysiology) [9,36]. As such, the application of the SIR-Profile takes into consideration the overall magnitude (Δ pre- to post-exercise) and interactions between the cluster of inflammatory cytokines (i.e. TNFα, IL-1β, IL-6, IL-8, IL-10, IL-1ra) potentially links to pathophysiology of heat stroke; which is similar to the previously reported pro- to anti-inflammatory ratio [2,3,18,19,28]. A moderately positive correlation between SIR-Profile with M-T_re_ and Δ T_re_ was observed. The total variation in SIR-Profile can be explained by M-T_re_ and Δ T_re_, with 31.2% and 16.6% significant contributions, respectively. The adjunct analysis of cardiovascular strain as an identified confounding factor during the screening process provided insignificant contribution. These outcomes are likely due to the influence of IL-10 and IL-1ra within the SIR-Profile. However, in alternate and/or future states of greater systemic inflammatory responses as a result of exertional or exertional-heat stress, such a profile would be able to identify values indicative of clinical significance warranting close observation or attention [4,10].

Previous exploratory and case research has detailed Ex-GIS reported by individuals that experience substantial intestinal injury and systemic immune outcomes in response to exertional and exertional-heat stress, with more pronounced outcomes observed as a result of exercising in the heat [18,19,33,34]. It is, however, also recognized that rapid onset and transient Ex-GIS occurs due to gastrointestinal functional issues, including feeding intolerance with or without a compromised gastrointestinal tract [22,23,34,67,68]. The low Ex-GIS incidence and severity rates in the current study (Table 2) are likely to primarily be due to EIGS circulatory-gastrointestinal pathway, since no pre- or during-exercise feeding intervention/s were employed in the included analyzed metadata, and thus, it is unlikely that the EIGS neuroendocrine-gastrointestinal pathway with or without feeding intolerance contributed substantially to the observed Ex-GIS. Nevertheless, a surprising set of findings of the current study were the moderate to weak positive correlations between Ex-GIS (i.e. total-, upper-, and lower-GIS) with M-T_re_ and Δ T_re_. For example, the variation in total-GIS can be explained by M-T_re_ and Δ T_re_, with 12.4% and 10.4% significant contributions, respectively; and the total variation in upper-GIS can be explained by M-T_re_ and Δ T_re_, with 11.8% and 10.5% significant contributions, respectively. These findings suggest Ex-GIS incidence and severity are exacerbated with increasing core body temperature, which are more likely to occur when exercise is performed in the heat.

From a professional practice perspective, it appears evident that heat mitigation strategies (e.g. heat acclimation, pre- and/or per-cooling strategies) that attenuate the change and/or maximum core body temperature may be effective for reducing exercise-associated perturbations to gastrointestinal integrity, systemic responses, Ex-GIS, and reducing the risk of developing any health and/or performance implications linked to EIGS and Ex-GIS. Previous research exploring internal or external pre- and/or pre-cooling, and heat acclimation protocols [20,69–72] have however shown modest to no beneficial outcomes to EIGS biomarkers, dependent on the aggressiveness of heat acclimation protocol and cooling intervention. These null outcomes from intervention studies are likely due to confounder control methodological concerns and the lack of sufficient exertional-heat stress and variable response, as highlighted in Costa et al. [10]. This area of investigation is in its infancy stage and warrants substantial exploration.

In conclusion, the current study provides evidence that 2 h of strenuous endurance exercise, with and/or without heat exposure, promotes increases in gastrointestinal integrity, bacterial endotoxin lumen to circulation translocation, systemic inflammatory cytokine responses, and Ex-GIS. Significant strong to moderate correlations were observed between M-T_re_ and Δ T_re_ with plasma concentrations of I-FABP, sCD14, and anti-inflammatory cytokines; and moderate to weak positive correlations with Ex-GIS. M-T_re_ and Δ T_re_ can in part predict the magnitude of increase in intestinal epithelial cell injury, bacterial endotoxin translocation, anti-inflammatory cytokine responses, and Ex-GIS.

## Highlights

- Maximal and change in core body temperature, in response to exercise with and without heat exposure, can predict the magnitude of intestinal epithelial injury, bacterial endotoxin translocation into systemic circulation, and systemic inflammatory responses.
- Maximal and change in core body temperature, in response to exercise with and without heat exposure, can predict the magnitude of exercise-associated gastrointestinal symptoms, namely upper gastrointestinal symptoms.

## Abbreviation

Δ T_re_: change in rectal temperature
EIGS: exercise-induced gastrointestinal syndrome
EndoCAb: Endogenous endotoxin core antibody
Ex-GIS: exercise-associated gastrointestinal symptoms
GIS: gastrointestinal symptoms
I-FABP: intestinal fatty acid protein
IgM: immunoglobulin M
LBP: lipopolysaccharide binding protein
sCD14: soluble CD14
SIR: systemic inflammatory profile
T_re_: rectal temperature
M-T_re_: maximum rectal temperature

## Supplementary Material

Supplemental Material
