# Supplementary material for: The increase in core body temperature in response to exertional-heat stress can predict exercise-induced gastrointestinal syndrome
Source: Temperature (Austin). 2023 May 24;11(1):72–91. doi: 10.1080/23328940.2023.2213625 (PMC10989703; doi:10.1080/23328940.2023.2213625)
Supplement: Supplemental Material [file KTMP_A_2213625_SM3091.docx]

**Supplementary Table 1.** Monash University Human Research Ethics Committee research ethics approval numbers of studies inclusive of exertional and exertional-heat stress experimental trials.

|  |  |  |  |
| --- | --- | --- | --- |
| **Study reference** |  | **Ethics approval number** | **Approval date** |
|  |  |  |  |
|  |  |  |  |
| Gaskell et al., 2020 | [13] | 2017-8847 | 28/02/2018 |
| Russo et al., 2021 | [15] | 12799 | 09/04/2018 |
| Russo et al., 2021 | [16] | 12799 | 09/04/2018 |
| Russo et al., 2021 | [17] | 12799 | 09/04/2018 |
| Snipe et al., 2018 | [18] | CF13/3647- 2013001878 | 10/09/2014 |
| Snipe et al., 2018 | [19] | CF13/3647- 2013001878 | 10/09/2014 |
| Snipe & Costa 2018 | [20] | CF15/3845- 2015001683 | 30/11/2015 |
| Snipe et al., 2017 | [27] | CF13/3647- 2013001878 | 10/09/2014 |
| Costa et al., 2023 | [36] | 29581 | 06/07/2021 |
|  |  |  |  |
